# Lipocalin 2 stimulates bone fibroblast growth factor 23 production in chronic kidney disease

**DOI:** 10.1038/s41413-021-00154-0

**Published:** 2021-08-02

**Authors:** Guillaume Courbon, Connor Francis, Claire Gerber, Samantha Neuburg, Xueyan Wang, Emily Lynch, Tamara Isakova, Jodie L. Babitt, Myles Wolf, Aline Martin, Valentin David

**Affiliations:** 1 Division of Nephrology and Hypertension, Department of Medicine, and Center for Translational Metabolism and Health, Institute for Public Health and Medicine, Northwestern University Feinberg School of Medicine, Chicago, IL USA; 2 Nephrology Division, Program in Membrane Biology, Massachusetts General Hospital, Harvard Medical School, Boston, MA USA; 3 Division of Nephrology, Department of Medicine, and Duke Clinical Research Institute, Duke University School of Medicine, Durham, NC USA

**Keywords:** Multihormonal system disorders, Multihormonal system disorders, Calcium and vitamin D

## Abstract

Bone-produced fibroblast growth factor 23 (FGF23) increases in response to inflammation and iron deficiency and contributes to cardiovascular mortality in chronic kidney disease (CKD). Neutrophil gelatinase-associated lipocalin (NGAL or lipocalin 2; LCN2 the murine homolog) is a pro-inflammatory and iron-shuttling molecule that is secreted in response to kidney injury and may promote CKD progression. We investigated bone FGF23 regulation by circulating LCN2. At 23 weeks, Col4a3^KO^ mice showed impaired kidney function, increased levels of kidney and serum LCN2, increased bone and serum FGF23, anemia, and left ventricular hypertrophy (LVH). Deletion of *Lcn2* in CKD mice did not improve kidney function or anemia but prevented the development of LVH and improved survival in association with marked reductions in serum FGF23. *Lcn2* deletion specifically prevented FGF23 elevations in response to inflammation, but not iron deficiency or phosphate, and administration of LCN2 increased serum FGF23 in healthy and CKD mice by stimulating *Fgf23* transcription via activation of cAMP-mediated signaling in bone cells. These results show that kidney-produced LCN2 is an important mediator of increased FGF23 production by bone in response to inflammation and in CKD. LCN2 inhibition might represent a potential therapeutic approach to lower FGF23 and improve outcomes in CKD.

## Introduction

Bone production of fibroblast growth factor 23 (FGF23) is increased in patients and animals with chronic kidney disease (CKD)^1–3^ and is associated with the development of left ventricular hypertrophy (LVH), heart failure, and mortality.^1,2,4–7^ Excess circulating FGF23 is the first major perturbation of mineral metabolism that occurs in CKD, however, the complex mechanisms that trigger elevations of FGF23 in CKD remain incompletely understood. Among these, multiple studies showed contributions of inflammation,^8^ iron deficiency,^9^ anemia,^2^ and local osteocyte defects.^1^ Notably, circulating FGF23 levels increase as kidney disease progresses, suggesting that kidney-bone crosstalk may contribute to excessive production of FGF23 by bone in response to kidney injury.^10,11^

Lipocalin 2, (LCN2) also known as neutrophil gelatinase-associated lipocalin in humans (NGAL) is a 25 kD lipophilic glycoprotein member of the lipocalin superfamily^12^ involved in innate immunity. The established role of LCN2 is to limit bacterial growth by binding to bacterial siderophores, which are low molecular weight chelators of ferric iron that are produced by bacteria to scavenge iron from their surrounding environment. In addition, LCN2 functions as an iron transporter by binding mammalian siderophores,^13,14^ and stabilizes labile iron/siderophore complexes.^15,16^ LCN2 allows cells to tolerate supra-physiological iron concentrations by scavenging free iron^17–19^ and protects against labile iron-mediated cytotoxicity. LCN2 is secreted by various cell types and tissues, including but not limited to immune cells,^20^ bone,^21^ liver,^22^ intestines,^23^ heart^24^ and kidney,^25^ and its expression is regulated mainly by infection and inflammatory status.

In patients with acute and CKD, kidney production of NGAL/LCN2 increases and can be detected in the urine and plasma; elevated urinary NGAL/LCN2 is a biomarker of acute kidney injury (AKI).^25^ Increased kidney *LCN2* expression in AKI is thought to be a component of the systemic inflammatory response to AKI that helps redirect iron to support repair of renal tubular cells.^26^ In CKD, kidney expression and urine and serum LCN2 levels are also elevated, presumably in response to chronic kidney injury, inflammation, and infiltrating cells.^27–30^ Studies in which *Lcn2* genetic deletion delayed CKD progression in mice demonstrate that LCN2 is not only a biomarker but could be a potential driver of CKD progression.^31^ Despite the links between LCN2 regulation and iron homeostasis, inflammation, and kidney disease, each of which is also involved in FGF23 regulation, potential direct relationships between LCN2, FGF23 regulation, and FGF23-associated outcomes have not been studied.

In the present study, we propose a novel mechanism to explain coincident increases in LCN2 and FGF23 soon after kidney injury,^32^ and the strong independent association between elevated levels of FGF23 and inflammatory markers.^33^ We hypothesized that bone is a target of kidney-secreted LCN2 and that increased LCN2 stimulates bone production of FGF23 in CKD. To test our hypothesis, we investigated the role of LCN2 in FGF23 regulation in health and in CKD. We show that circulating levels of LCN2 increased and paralleled CKD progression in the Col4a3^KO^ mouse model of CKD, and that kidney was the organ with the highest expression of *Lcn2* in CKD. We further show that genetic deletion of LCN2 in mice that develop CKD prevented increases in bone and circulating levels of FGF23 and development of LVH, and improved lifespan, despite CKD and anemia of unchanged severity. Finally, we show that increased circulating LCN2 stimulates *Fgf23* transcription through stimulation of cyclic AMP-mediated signaling in bone cells.

## Results

### Increased serum NGAL is associated with excess FGF23 in patients with CKD

**Fig. 1:**
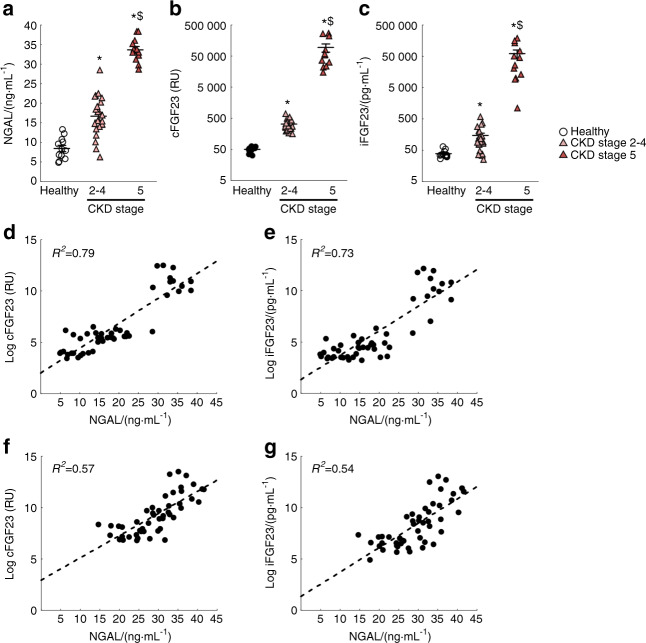
FGF23 and NGAL levels increase in patients with CKD. Levels of serum (**a**) NGAL, (**b**) total FGF23 (cFGF23), and (**c**) intact FGF23 (iFGF23) increase with the progression of kidney disease. NGAL correlates with both (**d**) and (**f**) cFGF23 (*R*^2^ = 0.79, partial correlation *R*^2^ = 0.57) and (**e**) and (**g**) iFGF23 (*R*^2^ = 0.73, partial correlation *R*^2^ = 0.54) levels, (**d**) and (**e**) unadjusted variables or (**f**) and (**g**) adjusted by eGFR. *P* values were determined by a two-sided, paired *t*-test. Values are mean ± SE, *n* ≥ 12/group, *P* < 0.05 vs. *Healthy, ^$^Stage 2–4

NGAL/LCN2 is a secreted pro-inflammatory and iron shuttling glycoprotein which might contribute to the progression of CKD.^31^ Given that FGF23 production is increased in response to inflammation and iron deficiency, we investigated whether FGF23 levels correlate with circulating NGAL. In serum collected from healthy volunteers and patients with CKD, we found that NGAL, cFGF23, and iFGF23 levels increased as kidney function declined (Fig. 1a–c). We further found that log(cFGF23) and log(iFGF23) strongly correlated with NGAL levels (log cFGF23 *R*^2^ = 0.79; log iFGF23 *R*^2^ = 0.73) and significantly associated with ascending NGAL levels (Fig. 1d, e). These associations remained significant after adjusting for kidney function [partial correlation log cFGF23 *R*^2^ = 0.57; partial correlation log iFGF23 *R*^2^ = 0.54] (Fig. 1f, g). Linear regression models showed that NGAL, independently of eGFR, was strongly associated with excess total and intact FGF23 (Supplementary Tables 1 and 2). Taken together, the strong eGFR-independent associations of NGAL/LCN2 with FGF23 levels suggest that NGAL/LCN2 might regulate FGF23 production in CKD.

### Col4a3^KO^ mouse model of CKD with increased Lipocalin 2

**Fig. 2:**
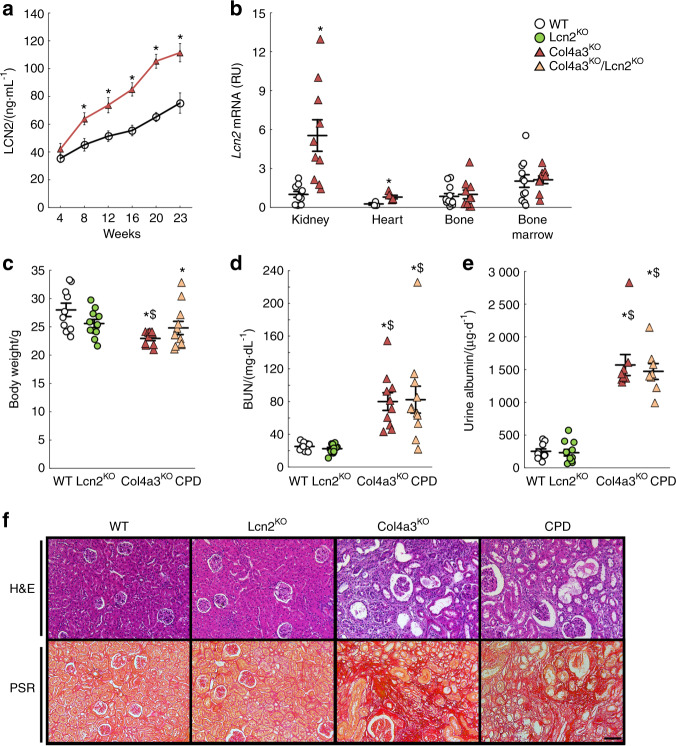
Increased production of lipocalin 2 does not contribute to impaired kidney function in the Col4a3^KO^ mouse model of chronic kidney disease. (**a)** Levels of serum lipocalin 2 (LCN2) measured in 4–23-week-old wild-type (WT) and Col4a3^KO^ mice. (**b)** Levels of Lcn2 mRNA expression (reported to Rpl19 expression, and set at 1 in WT kidneys) in kidney, heart, bone, and bone marrow from 23-week-old WT and Col4a3^KO^ mice. (**c)** Body weight, (**d**) blood urea nitrogen (BUN) levels, (**e**) 24 h urine albumin levels and (**f**) bright-field microscopy of hematoxylin & eosin (H&E) and picrosirius red (PSR, scale bar = 75 µm) stainings of kidneys from 23-week-old WT, Lcn2^KO^, Col4a3^KO^, and Col4a3^KO^/Lcn2^KO^ (CPD) mice. *P* values were determined by 2-sided, paired *t-*test. Data are presented as mean ± SE, *n* ≥ 5 per group, *P* < 0.05 vs.*WT, ^$^Lcn2^KO^

We reported that C57Bl6–Col4a3^KO^ mice experience progressive declines in renal function and develop LVH.^1,34^ Here, we report that circulating levels of LCN2 increased in an age-dependent manner in WT and Col4a3^KO^ mice, and were higher in Col4a3^KO^ mice with CKD than in WT mice (Fig. 2a). Lcn2 is one of the leading upregulated genes in kidneys from Col4a3^KO^ mice.^30^ In our current study, expression of *Lcn2* was highly elevated in the kidney, and to a lower extent in the hearts, but not in bone or bone marrow of 23-week-old Col4a3^KO^ mice with advanced CKD compared to WT mice (Fig. 2b). This suggests that LCN2 is secreted into the circulation mainly by the injured kidneys.

### Lipocalin 2 deletion does not improve renal function in Col4a3^KO^ mice

To assess the contribution of LCN2 to CKD progression and CKD-associated outcomes, we deleted *Lcn2* from WT and Col4a3^KO^ mice and studied the phenotype of WT, Lcn2^KO^, Col4a3^KO^, and mice with compound deletion of *Lcn2* and *Col4a3* (CPD: Lcn2^KO^/Col4a3^KO^) littermates. Col4a3^KO^ and CPD mice showed reduced body weight compared to WT and Lcn2^KO^ mice (Fig. 2c) but BUN and albuminuria were similar in Col4a3^KO^ and CPD mice (Fig. 2d, e). Similar degrees of glomerulosclerosis, tubular atrophy, and interstitial fibrosis were also recorded in Col4a3^KO^ and CPD mice (Fig. 2f). These results suggest that *Lcn2* deletion in CKD does not protect against loss of kidney function in the Col4a3^KO^ model.

### Lipocalin 2 deficiency does not prevent anemia of CKD

**Fig. 3:**
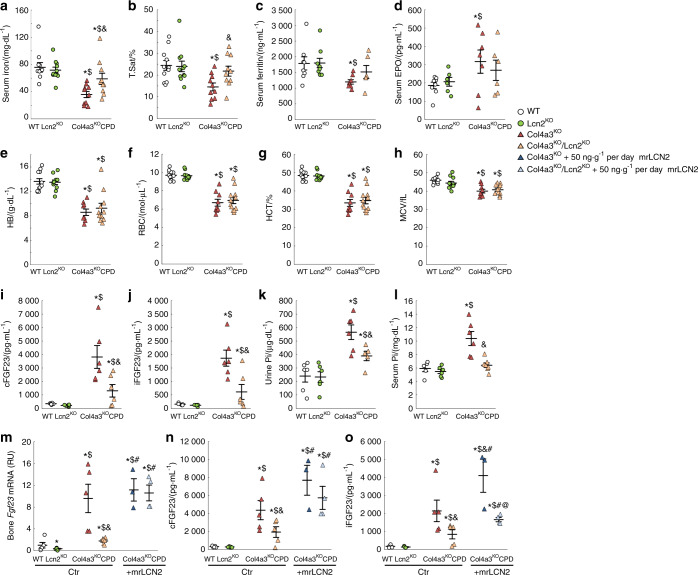
Increased lipocalin 2 contributes to the elevation of FGF23 in Col4a3^KO^ mice with advanced chronic kidney disease. Levels of (**a**) serum iron, (**b**) serum transferrin saturation, (**c**) serum ferritin, (**d**) serum erythropoietin, (**e**) hemoglobin (HB), (**f**) red blood cells (RBC), (**g**) hematocrit (HCT) and (**h**) mean corpuscular volume (MCV) measured in 23-week-old WT, Lcn2^KO^, Col4a3^KO^, and Col4a3^KO^/Lcn2^KO^ (CPD) mice. Levels of (**i**), (**n**) serum total FGF23 (cFGF23), (**j**), (**o**) serum intact FGF23 (iFGF23), (**k**) urine phosphate (Pi), (**l**) serum Pi, and (**m**) bone Fgf23 mRNA expression, measured in baseline (**a**)–(**l**) and control-treated (**m**)–(**o**) 23-week-old WT, Lcn2^KO^, Col4a3^KO^, and Col4a3^KO^/Lcn2^KO^ (CPD) mice, and in mouse recombinant lipocalin 2 (mrLCN2, 50 ng·g^−1^ per day)–treated Col4a3^KO^ and CPD mice (**m**)–(**o**). Data are presented as mean ± SE, *n* ≥ 3 per group, *P* < 0.05 vs.*WT, ^$^Lcn2^KO^, ^&^Col4a3^KO^, ^#^CPD, ^@^Col4a3^KO^ + mrLCN2

We next tested the hypothesis that LCN2 contributes to disturbed iron metabolism and erythropoiesis in CKD,^35,36^ given that one of the major functions of LCN2 is to transport iron. At 23 weeks, Lcn2^KO^ mice did not display changes in circulating iron or hematological parameters (Fig. 3a–h). Col4a3^KO^ animals showed decreased circulating iron, ferritin (as a measure of iron stores), hemoglobin, red blood cell number, hematocrit, and mean corpuscular volume consistent with the development of microcytic anemia (Fig. 3a–h).^2^ Deletion of *Lcn2* in Col4a3^KO^ mice partially corrected circulating iron, transferrin saturation, and ferritin levels, suggesting that increased levels of LCN2 contribute to disordered iron metabolism in CKD (Fig. 3a–c). Serum EPO levels were inappropriately low in both Col4a3^KO^ and CPD mice (Fig. 3d), and despite increases in circulating iron and iron stores, CPD mice showed a similar degree of microcytic anemia as Col4a3^KO^, assessed by reduced hemoglobin, red blood cell number, hematocrit and mean corpuscular volume (Fig. 3f–h).

### Deletion of Lipocalin 2 reduces FGF23 production in CKD

Total cFGF23 and iFGF23 levels were similar in Lcn2^KO^ and WT mice (Fig. 3i, j) at 23 weeks of age. This was accompanied by normal urine Pi excretion and serum phosphate (Fig. 3k, l). As previously reported, serum cFGF23 and iFGF23 levels were highly increased in Col4a3^KO^ mice with advanced CKD (Fig. 3i, j), which also showed hyperphosphatemia with increased urine Pi excretion (Fig. 3k, l). These changes were markedly attenuated by *Lcn2* deletion in Col4a3^KO^ mice, which demonstrated 60% reductions in serum cFGF23 and iFGF23, 80% reduction in bone *Fgf23* mRNA expression (Fig. 3i, j, m), and significantly reduced urine Pi excretion in CPD mice (Fig. 3k) and serum phosphate levels also decreased in CPD mice (Fig. 3l).

To further confirm that LCN2 directly regulates FGF23 in CKD, we administered murine recombinant LCN2 to Col4a3^KO^ and CPD mice for 8 weeks, from 16 to 23 weeks, using osmotic minipumps. Administration of LCN2 to Col4a3^KO^ mice did not further increase bone *Fgf23* mRNA or serum cFGF23 levels (Fig. 3m, n), but resulted in higher levels of serum iFGF23 (Fig. 3o). More importantly, LCN2 administration to CPD mice increased bone *Fgf23* mRNA and both serum cFGF23 and iFGF23 levels to similar levels observed in control Col4a3^KO^ mice (Fig. 3m–o), showing that increased LCN2 levels contribute to increased FGF23 production in CKD.

### Deletion of Lipocalin 2 prevents LVH and improves survival in CKD

**Fig. 4:**
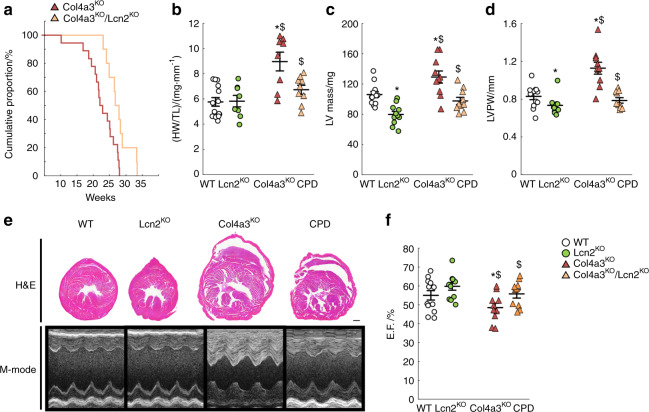
Lipocalin 2 deletion in Col4a3^KO^ mice improves survival and prevents the development of left ventricular hypertrophy. (**a**) Kaplan–Meier cumulative proportion of mice surviving. Both Col4a3^KO^ (red) and Col4a3^KO^/Lcn2^KO^ (coral) mice show reduced lifespan (*P* < 0.05 vs. WT), but Col4a3^KO^/Lcn2^KO^ survive longer than Col4a3^KO^ mice (*P* < 0.05 vs. Col4a3^KO^). (**b**) Heart weight to tibia length (HW/TL) ratio, and (**c**) left ventricular (LV) mass, (**d**) LV posterior wall (LVPW) thickness, (**e**) bright-field microscopy of hematoxylin & eosin staining (H&E) of heart cross-sections (scale bar = 1 mm), and M-mode echocardiography, and (**f**) ejection fraction (EF) measured in 23-week-old WT, Lcn2^KO^, Col4a3^KO^, and Col4a3^KO^/Lcn2^KO^ (CPD) mice. **c**, **d**, **f** are calculated from echocardiography analysis. Data are presented as mean ± SE, *n* ≥ 10 per group, *P* < 0.05 vs.*WT, ^$^Lcn2^KO^

In CKD, elevated FGF23 is associated with the development of LVH, heart failure, and death, and administration of FGF23 to WT mice induces LVH.^4^ As previously reported,^1,34^ C57Bl6-Col4a3^KO^ mice show a shortened lifespan and die at an average of 23 weeks of age (Fig. 4a). Development of LVH in C57Bl6-Col4a3^KO^ mice with advanced CKD may contribute to a shortened lifespan, as an increase in whole heart and left ventricular mass in Col4a3^KO^ mice eventually leads to impaired cardiac function (Fig. 4b–f), and rescue of LVH is associated with extended lifespan in this model.^1^ Despite a similar degree of CKD and anemia, *Lcn2* deletion improved survival, as CPD mice lived on average 3 weeks longer than Col4a3^KO^ mice (Fig. 4a). Indeed, histology and echocardiography analyses showed a lower heart weight to tibia length ratio, reduced LV mass, and posterior wall thickness in CPD vs. Col4a3^KO^ mice with advanced CKD, demonstrating that *Lcn2* deletion prevents the development of LVH in CKD (Fig. 4b–e). In addition, CPD mice showed higher ejection fraction (EF) than Col4a3^KO^ mice with CKD, suggesting that *Lcn2* deletion also preserves cardiac function (Fig. 4f), perhaps by limiting FGF23 production.

### Lipocalin 2 regulates bone FGF23 production in response to inflammation, but not to phosphate or iron deficiency

**Fig. 5:**
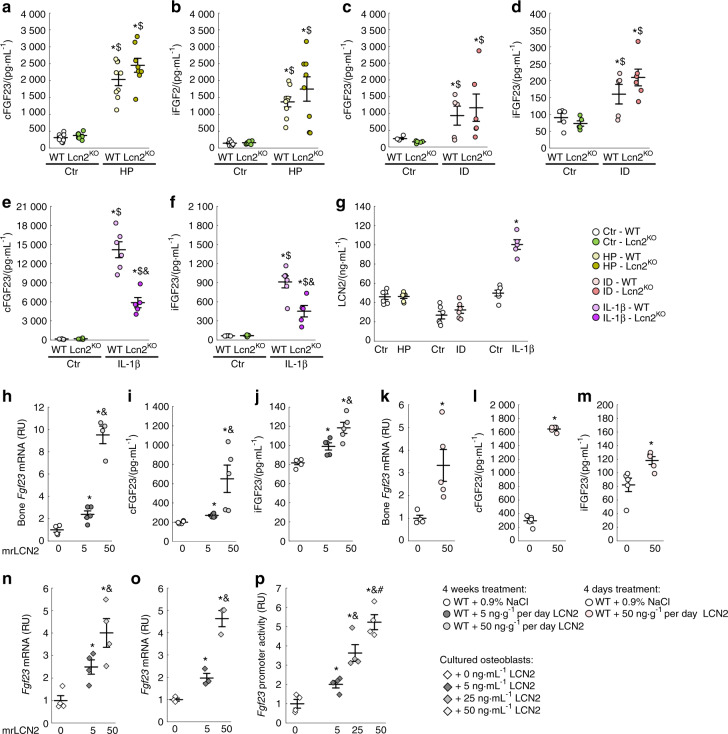
Lipocalin 2 mediates inflammation-induced FGF23 elevation. Levels of serum total FGF23 (cFGF23) and serum intact FGF23 (iFGF23) inWT and Lcn2^KO^ mice fed (**a**), (**b**) a 0.8% (Ctr) or a 2% high phosphate (HP) diet from 6 to 12 weeks, (**c**), (**d**) a 48 ppm (Ctr) or a 3 ppm iron deficient (ID) diet from 3 to 6 weeks, and (**e**), (**f**) 6 h post-injection of a single dose of saline (Ctr) or interleukin 1β (IL-1β, 250 ng·g^−1^) in 6-week old mice. (**g**) Serum levels of LCN2 in WT mice submitted to the same challenges. Data are presented as mean ± SE, *n* ≥ 5 per group, *P* < 0.05 vs.*Ctr-WT, ^$^Ctr-Lcn2^KO^, ^&^IL-1β-WT. Levels of (**h**), (**k**) bone Fgf23 mRNA, (**i**), (**l**) serum total FGF23 (cFGF23) and (**j**), (**m**) serum intact FGF23 (iFGF23) in WT mice treated continuously for 4 weeks with 0 (0.9%NaCl), 5 and 50 ng·g^−1^ of mouse recombinant LCN2 (mrLCN2) (**h**)–(**j**) or injected daily for 4 d with 0 (0.9%NaCl) and 50 ng·g^−1^ of mrLCN2 (**k**)–(**m**). Data are presented as mean ± SE, *n* ≥ 5 per group, *P* < 0.05 vs. *non treated, ^&^5 ng·g^−1^ mrLCN2. *Fgf23* mRNA levels (**n**) in primary osteoblast and (**o**) in MC3T3-E1 osteoblasts cultures treated with 0–50 osteoblasts cultures treated with 0–50 ng·mL^–1^ of mrLCN2. (**p**) *Fgf23* promoter activity in *Fgf23* promoter-reporter MC3T3-E1 osteoblast cultures treated with 0–50 ng·mL^−1^ of mrLCN2. Data are presented as mean ± SE, *n* ≥ 3 per group, *P* < 0.05 vs. *non treated, ^&^5 ng·mL^−1^, ^#^25 ng·mL^−1^ mrLCN2

Hyperphosphatemia, iron deficiency/anemia, and inflammation are potent stimuli of bone FGF23 production during CKD progression.^2,8^ We tested whether *Lcn2* deletion would alter FGF23 regulation by phosphate, iron, or inflammatory stimuli induced by dietary Pi loading, iron restriction, and acute IL1β injections, respectively. As expected, WT animals fed a high phosphate diet displayed higher levels of both serum cFGF23 and iFGF23 levels, and Lcn2^KO^ mice showed similar elevations of cFGF23 and iFGF23 on the high phosphate diet, suggesting that phosphate regulation of FGF23 is independent of LCN2 (Fig. 5a, b). As previously shown,^8^ WT mice fed a low iron diet also showed increased cFGF23 and mildly elevated iFGF23 levels. Despite the role of LCN2 as an iron transporter, Lcn2^KO^ mice showed similar increases in both serum cFGF23 and iFGF23 levels to WT mice in response to a low iron diet (Fig. 5c, d), suggesting that LCN2 regulation of FGF23 is iron-independent. Finally, consistent with previous reports,^8^ acute inflammation induced by administration of IL-1β to WT mice dramatically increased cFGF23 levels and, to a much lower extent, iFGF23 levels. However, Lcn2^KO^ mice showed a blunted response to IL-1β, as cFGF23 and iFGF23 elevations were reduced by ~50% in IL-1β-treated Lcn2^KO^ mice (Fig. 5e, f). This demonstrates that LCN2 partially mediates FGF23 production during inflammation (Fig. 5g).

### Lipocalin 2 directly regulates bone FGF23 production

To further understand whether LCN2 only potentiates the effects of inflammatory stimuli on FGF23 production in CKD or inflammation or whether LCN2 directly stimulates FGF23 production, we administered LCN2 to 6-week old WT mice either for 4 weeks using osmotic minipumps or during 4 days of repeated LCN2 injections. Continuous administration of LCN2 at low (5 ng·g^–1^ per day) or high doses (50 ng·g^–1^ per day), dose-dependently increased bone *Fgf23* mRNA expression, serum cFGF23 levels, and led to a mild increase in iFGF23 levels (Fig. 5h–j). Similarly, short-term intermittent administration of 50 ng/g/day LCN2 to C57Bl6 mice resulted in increased osseous *Fgf23* mRNA, higher circulating cFGF23 levels, and a mild increase in iFGF23 levels (Fig. 5k–m). In addition, 24 h of LCN2 treatment increased *Fgf23* transcription in cultured bone marrow stromal cells (BMSC) or MC3T3-E1 cell lines (Fig. 5n–o), and dose-dependently increased *Fgf23* promoter activity in MC3T3-E1 Fgf23-promoter-reporter cells (Fig. 5p). These combined results demonstrate that LCN2 increases FGF23 production in bone by stimulating *Fgf23* expression in osteoblasts and osteocytes.

### Lipocalin 2 stimulates bone FGF23 production through a cAMP-dependent mechanism

**Fig. 6:**
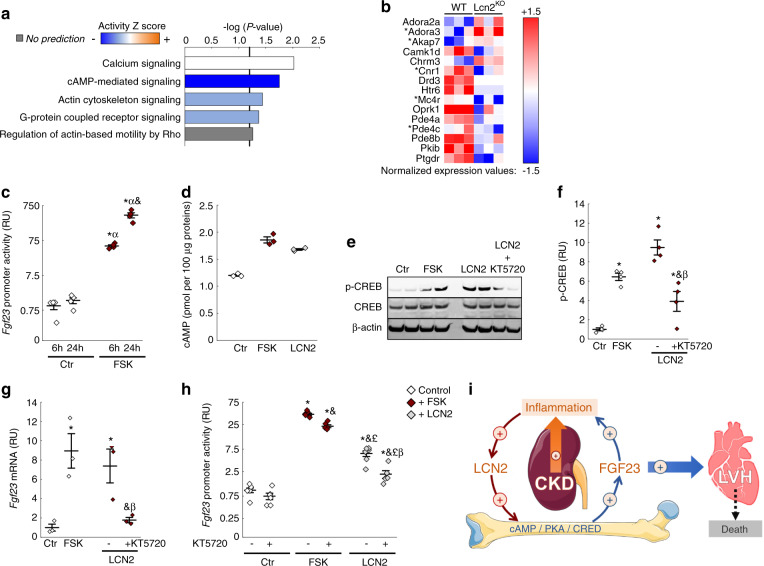
cAMP signaling mediates lipocalin 2-induced bone stimulation of FGF23 production in osteoblasts. RNA sequencing of WT and Lcn2KO cortical bone identified 614 differentially regulated genes in Lcn2KO compared to WT (cutoffs: *P* < 0.05 and absolute fold change of 2). (**a)** Top 5 canonical pathways identified by ingenuity pathway analysis from the 614 genes dataset. (**b)** Normalized expression of dataset genes involved in the cAMP pathway with a twofold cutoff and *P* < 0.05 with the exception of *which indicates *P* < 0.1. (**c)** Fgf23 promoter activity in Control (Ctr) or Forskolin (FSK) -treated Fgf23 promoter-reporter MC3T3-E1 osteoblast cultures. (**d)** cAMP levels in Ctr, FSK, and LCN2 -treated MC3T3-E1 osteoblast cultures. (**e)** Representative micrograph and quantification (**f**) of western blotting detection of phosphorylated CREB (p-CREB) and total CREB, normalized to β-actin, in protein extracts from MC3T3-E1 osteoblasts treated with Ctr, FSK, and LCN2, and co-treated with LCN2 and cAMP inhibitor KT5720. Effects of Ctr, FSK, and LCN2 -treatment and KT5720 co-treatment on Fgf23 mRNA in MC3T3 osteoblasts (**g**) and promoter activity (**h**) in Fgf23 promoter-reporter MC3T3-E1 osteoblast cultures. Data are presented as mean ± SE, *n* ≥ 3 per group, *P* < 0.05 vs.* 6 h-Ctr, α 24 h-Ctr, and 6 h-FSK, £ 6 h-FSK + KT5720, β 6 h-LCN2. (**i)** Progressive alterations in kidney morphology and function induce inflammation-dependent lipocalin secretion leading to increased circulating LCN2. In bone, LCN2 increases FGF23 production through a cAMP/PKA/CREB-dependent mechanism, which contributes to excess FGF23 in CKD. Elevated FGF23 exerts pro-inflammatory effects, aggravating the inflammatory status in CKD. Excess FGF23 also targets the heart and contributes to the development of cardiac disease and mortality

To understand the primary mechanisms leading to increased bone production of FGF23 in response to elevated LCN2, we performed RNA sequencing of cortical bone isolated from 12-week old WT and Lcn2^KO^ mice. We found that 614 genes were differentially expressed in Lcn2^KO^ compared to WT mice (cutoffs: *P* < 0.05, absolute fold change (FC) of 2). Of these, 369 genes were downregulated and 245 genes were upregulated. The top 50 most upregulated and downregulated genes are shown in Table 1. Of note, we found a net (−2-fold) albeit non-significant reduction in *Fgf23* (*P* = 0.12). Using ingenuity pathway analysis (IPA, QIAgen), we identified the canonical signaling pathways predicted to be significantly changed in the cortical bone of Lcn2^KO^ mice. Among these, the top five pathways predicted to be downregulated or upregulated, cAMP-mediated signaling was predicted to be the most inhibited pathway (Fig. 6a), based on the z-score activation of multiple genes. A total of ten genes in this pathway were dysregulated in Lcn2^KO^ mice (*P* < 0.05, FC2), and selective enrichment of this pathway (*P* < 0.1, FC2) identified five additional genes of cAMP signaling in the entire dataset (Fig. 6b). To test whether LCN2 regulates FGF23 production through activation of cAMP-mediated signaling, we first verified that forskolin (FSK), a known inducer of cAMP and cAMP-sensitive pathways, stimulated *Fgf23* promoter activity in MC3T3-E1 Fgf23-promoter-reporter cells (Fig. 6c). Then, we compared the effects of FSK and LCN2 treatment on cAMP activation and found that both FSK and LCN2 treatment increased intracellular cAMP levels (Fig. 6d) and phosphorylation of the cAMP response element-binding protein (CREB) in MC3T3-E1 osteoblast-like cells 6 h post-stimulation (Fig. 6e, f). Co-treatment of LCN2 stimulated cells with KT5720, an inhibitor of cAMP activation blocked the effects of LCN2 on CREB phosphorylation, suggesting that cAMP mediates the effects of LCN2. Finally, both FSK and LCN2 increased Fgf23 mRNA (Fig. 6g) and Fgf23 promoter activity (Fig. 6h) at 6 h, and their effects were partially blocked by co-treatment with the KT5720. These data demonstrate that stimulation of FGF23 production by LCN2 in osteoblasts is mediated at least in part by cAMP signaling and that LCN2 and FGF23 are part of a vicious cycle in CKD (Fig. 6i).

**Table 1:** Topmost up- and down-regulated genes in tibiae from Lcn2^KO^ vs. WT (*P* < 0.05)

Up-regulated				Down-regulated			
Gene ID	Gene symbol	*P* value	Fold change	Gene ID	Gene symbol	*P* value	Fold change
98752	Fcrla	0.046	10.8	18164	Nptx1	0.004	−35.4
213002	Ifitm6	0.043	10.3	207618	Zfp804b	0.043	−21.4
52614	Emr4	0.007	9.4	258844	Olfr1090	0.000	−21.0
12775	Ccr7	0.024	7.8	72276	1700025M24Rik	0.000	−16.4
54124	Cks1b	0.043	7.7	12049	Bcl2l10	0.006	−14.4
69816	Mzb1	0.039	7.4	236069	Gm13238	0.006	−14.1
208501	1810043H04Rik	0.004	7.3	404287	V1rd19	0.027	−13.2
225895	Taf6l	0.005	7.2	57890	Il17re	0.008	−12.6
76933	Ifi27l2a	0.025	7.2	236874	Gm14743	0.006	−12.4
12532	Cdc25c	0.018	6.9	13505	Dsc1	0.000	−12.3
17537	Meis3	0.001	6.7	26927	Foxl2	0.013	−12.2
328830	A530064D06Rik	0.046	6.4	20129	Rptn	0.038	−12.1
80733	Car15	0.011	6.2	215472	Gm4792	0.002	−12.0
69169	Faim3	0.044	6.2	259103	Olfr616	0.042	−11.9
106757	Catsperd	0.008	6.1	320590	Svopl	0.003	−11.6
18003	Nedd9	0.020	6.0	74928	4930467K11Rik	0.001	−11.6
246787	Slc5a2	0.014	5.9	257975	Olfr598	0.027	−11.4
22067	Trpc5	0.018	5.9	216166	Plk5	0.011	−11.3
69456	Commd10	0.003	5.7	68304	Kdelc2	0.031	−11.2
100861742	Gm21179	0.005	5.5	258135	Olfr597	0.016	−10.6
240755	4933406M09Rik	0.015	5.5	20958	Sycp1-ps1	0.000	−10.5
100041694	Gm10451	0.003	5.4	319215	4932413F04Rik	0.000	−10.4
382522	Hist3h2bb-ps	0.012	5.3	17897	Myl3	0.045	−10.3
70952	Poteg	0.002	5.3	93762	Smarca5	0.039	−10.3
20305	Ccl6	0.024	5.1	100038621	Gm10839	0.003	−10.1

## Discussion

Excess FGF23 is associated with adverse outcomes in patients with CKD.^6,37,38^ There is a tight correlation between progression of CKD and FGF23, as FGF23 increases as kidney function declines, suggesting that molecules secreted by the kidney might regulate FGF23 production in CKD. Here, we identified LCN2, as a kidney messenger that targets the bone cells to increase FGF23 production. We show that excess LCN2 in CKD contributes to excess circulating FGF23, development of cardiac disease, and premature death, and that genetic ablation of Lcn2 in CKD partially reduces Fgf23 bone mRNA expression, circulating FGF23 levels and results in marked improvement in cardiac function and lifespan. We also found that LCN2 mediates the expression of *Fgf23* in response to inflammation and that LCN2 directly stimulates FGF23 in bone cells via activation of cAMP-mediated signaling.

LCN2 is primarily a bacteriostatic agent,^39^ that binds to hydrophobic ligands such as iron siderophores produced by bacteria.^40,41^ LCN2 excess is observed in multiple aseptic pathologies of inflammatory nature,^42,43^ and as such, LCN2 has become increasingly relevant in recent years as a potential clinical biomarker in inflammatory diseases.^44–46^ Most importantly, blood and urinary levels of LCN2 have been extensively studied as potential biomarkers for an early diagnosis of AKI and for monitoring of CKD severity. Transcriptome and proteomic studies identified LCN2 to be one of the most upregulated genes and one of the most highly induced proteins in the kidney in animal models of AKI^47,48^ and CKD.^30^ Beyond its status as a biomarker and early predictor of AKI, data also suggested that LCN2 could serve as a biomarker in CKD.^27^ In a previous study, LCN2 was shown to lead to progressive renal failure^31^ and *Lcn2* genetic deletion protected against CKD progression in 3/4 nephrectomized mice or jck mice with polycystic kidney disease. In contrast, we report that the severity of kidney disease was similar in the Col4a3^KO^ mouse model of progressive CKD, with and without global deletion of Lcn2. It is possible that *Lcn2* deletion might delay the onset of CKD, but at the advanced stage of CKD in our model, differences in kidney function and morphology following *Lcn2* deletion would be barely perceptible. In line with our results, Lcn2 deletion in the severe acute tubular necrosis model did not rescue kidney morphology and function.^49^

Deletion of LCN2 leads to increased circulating iron in Col4a3^KO^ mice. Prior studies showed that LCN2 was a key factor in the regulation of erythrocyte growth due to its ability to inhibit the maturation of bone marrow erythroid precursors^50^ or inhibition of erythropoiesis through induction of apoptosis.^50,51^ However, we found that the role of LCN2 in anemia of CKD was limited. Indeed, hemoglobin and hematocrit were similar in Col4a3^KO^ and in CPD mice, which is consistent with studies showing no significant correlations between LCN2 and the levels of hemoglobin, hematocrit, and erythrocytes in patients on hemodialysis.^52^

Iron deficiency, hyperphosphatemia, and inflammation contribute to FGF23 production during CKD.^1,8,9^ Whether the effects of Lcn2 deletion on FGF23 production in CKD are partially mediated by the increase in circulating iron or decrease in circulating phosphate that we observed in CPD mice requires further study, but the lack of effects of LCN2 deletion on FGF23 regulation in diet-induced iron deficiency strongly suggests an iron independent effect. Similarly, the lack of effects of LCN2 on FGF23 in a model of diet-induced hyperphosphatemia suggests that LCN2 does not play a role in phosphate-induced FGF23 production. Alternatively, LCN2 may constitute a mediator of the stimulatory effects of inflammation on FGF23 production. Lcn2 deletion in unchallenged WT mice only resulted in a trend toward reduced osseous *Fgf23* mRNA both by PCR and RNAseq without significant reduction in FGF23 levels. However, *Lcn2* deletion attenuated the increase in serum FGF23 levels induced by inflammatory stimuli. We previously showed that inflammation stimulates *Fgf23* transcription in the bone which results in increased serum cFGF23 levels with only mild elevations in circulating levels of biologically active iFGF23 levels due to coupled activation of FGF23 cleavage mechanisms.^8^ Consistent with these effects,^8^ LCN2 administration mainly increased *Fgf23* transcription and secretion of cFGF23 and had a very modest impact on serum iFGF23 levels in the non-CKD models in the present study. However, LCN2 administration showed more pronounced effects on iFGF23 levels in Col4a3^KO^ mice with CKD, confirming that impaired FGF23 cleavage contributes to elevated serum iFGF23 levels in CKD.^8,53,54^

Interestingly, LCN2 directly targets bone cells and induces activation of cAMP/PKA/CREB signaling in osteoblasts. Previous studies have shown that LCN2 also activates cAMP signaling in the brain and spermatozoa,^21,55^ supporting cAMP signaling as a specific mediator of LCN2 effects. In bone, cAMP is also an important mediator of PTH effects^56,57^ and we confirm that cAMP-mediated signaling is a potent regulator of FGF23 transcription.^58^ The fact that cAMP signaling is regulated by both PTH and LCN2 upstream of FGF23 production suggests that cAMP-mediated signaling may play a central role in the regulation of FGF23 in CKD. Nevertheless, the overall impact of LCN2 on FGF23 circulating levels is highlighted by the independent relationship between FGF23 and NGAL in patients with CKD.

Excess FGF23 during CKD progression is associated with cardiovascular mortality via direct and reversible effects of FGF23 on cardiac myocytes that lead to the development of LVH.^4,5,59–61^ In the present study, inhibition of FGF23 production by genetic deletion of Lcn2 may have prevented the development of LVH and increased survival in Col4a3^KO^ mice with CKD. We previously showed in Col4a3^KO^ mice with CKD that overexpression of a bone matrix protein, DMP1, or treatment with ferric citrate, an iron-based phosphate binder, reduces FGF23 production and cardiovascular disease. This is the third study from our group showing that FGF23 reduction in CKD using different approaches, consistently leads to improvement of cardiac morphology and function despite persistent kidney disease.^1,2^ Ours study also suggests that LCN2 may be involved in secondary outcomes associated with progressive CKD, including the development of heart disease. Whether these effects are only mediated by FGF23 or whether there may also be some contribution from other known and possibly independent cardiac effects of LCN2,^24,62^ warrants additional studies which were beyond the scope of the present manuscript. Nonetheless, taken together our results emphasize the need for future studies focusing on FGF23 reduction in CKD.

To conclude, we showed that increased circulating LCN2 minimally contributes to iron deficiency in CKD, and does not have a determinant role on anemia of CKD, but mediates the stimulatory effects of inflammation on FGF23 production in bone by activating cAMP-mediated signaling, and contributes to the development of cardiac hypertrophy and mortality, likely through stimulation of FGF23 production. Since FGF23 also exerts pro-inflammatory effects,^30,63^ and strong correlations are observed in CKD between FGF23 and inflammatory markers,^33^ this represents a feed-forward loop, in which the effects of inflammation and FGF23 are fueling one another (Fig. 6i), offering one possible explanation for the maladaptive and exponential increases in FGF23 levels that occur in advanced CKD. Therefore, serum LCN2 emerges as a major kidney-bone crosstalk molecule that links the inflammation of kidney disease to FGF23 secretion from the bone and the development of cardiac disease during CKD.

## Materials and methods

### Human subjects

We used stored serum samples from 12 healthy volunteers and 36 patients with CKD stages 2–5 who participated in previous IRB-approved physiologic studies. All participants provided written informed consent to have their samples stored for future use for analysis of biomarkers related to kidney function and mineral metabolism. We measured NGAL using the NGAL ELISA assay (Abcam, Cambridge, MA, USA). We used a human intact FGF23 (iFGF23) enzyme-linked immunosorbent assay (ELISA) to measure the active iFGF23 protein and a C-terminal FGF23 (cFGF23) ELISA that recognizes the full-length protein and its C-terminal cleavage fragments to measure total FGF23 (both from Immutopics, Carlsbad, CA, USA).

### Animals

Heterozygous C57Bl6/J-LCN^+/−^, expressing a neo-cassette targeting a 1.9 kb fragment in exons 2–4,^39^ obtained from Dr. Shizuo Akira at Osaka University were crossed to generate LCN2^+/+^ (WT) and LCN2^−/−^ (Lcn2^KO^) mice. To assess the effects of acute inflammation, 12-week-old WT and Lcn2^KO^ mice have injected *ip* with 250 ng·g^−1^ of recombinant murine interleukin 1 beta (IL-1β, Cell signaling). All diets were manufactured by Teklad (Envigo, Dever, CO, USA). Mice were maintained on a standard diet (Teklad 7012) except when otherwise specified. True iron deficiency was induced by feeding 3-week-old mice an iron deficient diet (TD.80396) or a mineral adjusted control diet (TD.80394) for 3 weeks. WT and Lcn2^KO^ mice were fed a 2% high phosphate diet (TD.160039) or a mineral adjusted control diet (TD.80394) from 6 to 12 weeks of age. Six-week-old C57Bl6/J WT mice have injected ip during four days with murine recombinant LCN2 (50 ng·g^−1^ per day, 1857-LC-050, R&D systems, Minneapolis, MN, USA). Six-week-old C57Bl6/J WT mice were implanted ip with Alzet osmotic minipumps (Model 1004, Alzet, Cupertino, CA, USA) for 4 weeks to deliver murine recombinant LCN2 (5 or 50 ng·g^−1^ per day).

Heterozygous C57Bl6/J-Col4a3^tm1Dec^ mice were crossed to LCN2^+/−^^39^ mice to generate C57Bl6/J- Col4a3^+/+^LCN2^+/+^ (WT), Col4a3^+/+^LCN2^−/−^ (Lcn2^KO^), Col4a3^−/−^LCN2^+/+^ (Col4a3^KO^) and Col4a3^−/−^LCN2^−/−^ (CPD). We harvested samples on a set of 23-week-old male littermates. We recorded body weight at sacrifice. In a separate set of animals, we recorded the age of death on Col4a3^KO^ and CPD littermates to assess effects on lifespan. Col4a3^KO^ and CPD mice have implanted subcutaneously with Alzet osmotic minipumps for 8 weeks to deliver 50 ng·g^−1^ per day of murine recombinant LCN2. All studies were approved by Institutional Animal Care and Use Committee at Northwestern University.

### Biochemistry of mouse samples

We collected overnight urine samples from fasted animals housed overnight in metabolic cages and serum samples by intracardiac exsanguination. We used a murine intact FGF23 (iFGF23) enzyme-linked immunosorbent assay (ELISA) to measure the active iFGF23 protein and a C-terminal FGF23 (cFGF23) ELISA that recognizes the full-length protein and its C-terminal cleavage fragments to measure total FGF23 (both from Immutopics, Carlsbad, CA, USA). Phosphate, blood urea nitrogen (BUN), albumin, iron, and transferrin saturation were measured using colorimetric assays (Pointe Scientific, Canton, MI, USA). We measured serum ferritin using mouse ELISA assays (Alpco, Salem, NH, USA), circulating LCN2 using LCN2 mouse ELISA assay (Abcam, Cambridge, MA, USA), and erythropoietin using mouse EPO quantikine ELISA kit (R&D Systems, Minneapolis, MN, USA).

### RNA isolation, RT-PCR, and RNA sequencing

We isolated total RNA from tissues at sacrifice and from cell cultures using TRI reagent (Waltham, MA, USA) and purified RNA using RNeasy kit (Qiagen, Germantown, MD, USA).

For RNA sequencing, the total RNA library for each individual tibia was prepared using the TruSeq Total RNA-Seq Library Preparation Kit (Illumina, San Diego, CA), and the bar-coded cDNA libraries were sequenced for 75 bp single reads on one lane of Illumina NexSeq to generate a minimum of 100 million reads/library. Reads from each library were mapped to the mouse transcriptome and genome (UCSC mm10), filtered using StrandNGS software suite (Strand Life Sciences, Bangalore, Karnataka, India), and following Strand alignment and filtering pipelines. Reads were normalized using DESeq and we used baseline transformation to the median for each sample. FC and *P* value were calculated using moderated *T*-test and data were used for subsequent downstream pathway analyzes using the Ingenuity Pathway Analysis platform (IPA, Qiagen).

For RT-PCR, we synthesized first-strand cDNA (iScript cDNA Synthesis Kit, Bio-Rad Laboratories, Hercules, CA) and used the iCycler iQ real-time PCR detection system, iQ SYBR Green supermix (Bio-Rad Laboratories, Hercules, CA), and adequate primer pairs for real-time quantitative PCR analysis. The threshold of detection of each gene expression was set at optimal reaction efficiency. The expression was plotted against a standard dilution curve of relative concentration, normalized to 60S ribosomal protein L19 (*Rpl19*) expression in the same sample, and expressed as fold change versus respective controls.

### Echocardiography

We performed echocardiography under isoflurane anesthesia 1 week prior to sacrifice (at 22 weeks of age) using a Vevo 770 High-Resolution Imaging System (VisualSonics, Toronto, ON, Canada). We used the parasternal short- and long-axis views to obtain two-dimensional and M-mode images as previously described.^1,2,34^

### Hematologic analysis

Hematologic parameters were acquired in whole blood using the HEMAVET 950 hematology system (Drew Scientific Inc., Oxford, CT, USA) and analyzed with multispecies software using mouse settings as previously described.^2^

### Histology

Heart weight and tibia length were measured post sacrifice. Kidneys and hearts were collected at sacrifice, fixed in 100% ethanol, and embedded in paraffin. We collected 5-µm-thick sections using a rotary microtome. For analysis of the cardiac phenotype, we used cross-sections from the mid-chamber of the heart. We stained the sections with hematoxylin and eosin (H&E) to determine renal and cardiac morphology, picrosirius red (PSR) to determine kidney fibrosis. Images were acquired using light microscopy (Leica Microsystems, Buffalo Grove, IL, USA).

### Cell cultures and assays

We cultured MC3T3-E1 osteoblastic cell lines (ATCC) according to American type culture collection guidelines. We prepared BMSCs from 6-week-old mice according to a previously described protocol.^64^ We maintained MC3T3-E1 and BMSCs in α-MEM containing 10% FBS, 10 U·mL^–1^ penicillin, and 100 μg·mL^–1^ streptomycin. For all experimental conditions, we plated MC3T3-E1 at 3 × 10^4^ cells per well and BMSCs at 10 × 10^4^ cells per well and cultured for 3 weeks in osteoblast-differentiating medium (α-minimal essential medium, 10% fetal bovine serum, 10 U·mL^–1^ penicillin, 100 μg·mL^–1^ streptomycin, 10 mmol·L^–1^ β-glycerophosphate, and 50 μg·mL^–1^ ascorbic acid; Sigma–Aldrich, St Louis, MO) prior to treatment and collection. To assess *Fgf23* promoter activity, MC3T3-E1 cells were stably transfected with the pLuc-*Fgf23* promoter plasmid carrying a secreted luciferase expression cassette under the control of the proximal *Fgf23* promoter, a secreted alkaline phosphatase (SEALP) under the control of the CMV promoter, and a puromycin resistance cassette (Genecopoeia, Rockville, MD) as previously described.^1,8^ At day 21, cells were treated with recombinant murine 5–50 ng·mL^–1^ LCN2 (R&D systems), 10 μmol·L^–1^ FSK (Sigma Aldrich, St. Louis, MO, USA) and 12 μmol·L^–1^ KT5720 (Abcam). For promoter activity assays, Optimem medium containing 1% FBS 10 U•mL^–1^ penicillin and 100 μg·mL^–1^ streptomycin, supplemented with LCN2, FSK, and/or KT5720 was used for the last 24 h of culture. Promoter activity is represented by a relative luciferase unit normalized to pSEALP-CMV control. We conducted all experiments in triplicate.

### Protein assays and SDS-Page

MC3T3/E1 osteoblast cells grown in the osteogenic medium were treated for 6 h with FSK, LCN2, or LCN2 + KT5720 in Opti-MEM with 1% FBS. Protein extracts were prepared using T-Per lysis buffer (Thermo Fisher Scientific, MA, USA) containing protease inhibitors cocktail and immunoblots were performed as previously described.^2,8^ For SDS-Page, 100 µg of total proteins were loaded on a 4-to-12% Bis-Tris (midi) Gel (Invitrogen). CREB expression was detected using rabbit antibodies against phosphorylated CREB (1:1 000; 9198) and CREB (1:1 000; 9197) (Cell Signaling Technology, MA, USA) and goat anti-β Actin (1:1 000, 8229) (Abcam, MA, USA). Cyclic AMP accumulation in cells was measured using a cAMP direct immunoassay (Abcam) from protein extracts in 100 µL of 0.1 mol·L^–1^ HCl.

### Statistics

Data are presented as mean ± SEM. Univariate and multiple regression, ANOVA followed by Fisher and two-sided, paired *t* tests were used for statistical inference using Statistica software (Statsoft, OK, USA). *P* values < 0.05 were considered statistically significant.

### Study approval

All human participants studies provided written informed consent to have their samples stored for future use for analysis of biomarkers related to kidney function and mineral metabolism and were enrolled in IRB-approved physiologic studies. All animal studies were approved by Institutional Animal Care and Use Committee at Northwestern University.

## Supplementary information

Supplementary information

## Data Availability

All data associated with this study are present in the paper. Materials and protocols are available upon demand.
