# Supplementary material for: Lipocalin 2 stimulates bone fibroblast growth factor 23 production in chronic kidney disease
Source: Bone Res. 2021 Aug 2;9:35. doi: 10.1038/s41413-021-00154-0 (PMC8326281; doi:10.1038/s41413-021-00154-0)
Supplement: Supplementary file 1 — Supplementary information [file 41413_2021_154_MOESM1_ESM.docx]

## Supplementary Table 1: Multiple regression of serum log cFGF23 as a function of serum NGAL and GFR in patients with CKD.

|  | \| B1 \| \| --- \| | \| Std.Err.of B1 \| \| --- \| | \| b \| \| --- \| | \| Std.Err.of b \| \| --- \| | \| t(45) \| \| --- \| | \| p \| \| --- \| |
| --- | --- | --- | --- | --- | --- | --- | --- | --- | --- | --- | --- | --- |
| \| Intercept \| \| --- \| |  |  | 2.938 | 0.877 | 3.351 | 0.001638 |
| \| GFR/creatinine \| \| --- \| | -0.122 | 0.103 | -0.010 | 0.008 | -1.180 | 0.244266 |
| \| NGAL (ng/mL) \| \| --- \| | 0.797 | 0.103 | 0.217 | 0.028 | 7.732 | 0.000000 |

*Multiple regression using log cFGF23 as dependent variable as a function serum NGAL and GFR in 48 patients with Chronic Kidney Disease. Model significant at p=2.8E^-16^, R=0.89, R^2^=0.80, Adjusted R^2^=0.79.*

## Supplementary Table 2: Multiple regression of serum log iFGF23 as a function of serum NGAL and GFR in patients with CKD.

|  | \| B1 \| \| --- \| | \| Std.Err.of B1 \| \| --- \| | \| b \| \| --- \| | \| Std.Err.of b \| \| --- \| | \| t(45) \| \| --- \| | \| p \| \| --- \| |
| --- | --- | --- | --- | --- | --- | --- | --- | --- | --- | --- | --- | --- |
| \| Intercept \| \| --- \| |  |  | 1.344 | 1.024 | 1.312 | 0.196115 |
| \| GFR/creatinine \| \| --- \| | -0.001 | 0.118 | 0.000 | 0.010 | -0.006 | 0.995459 |
| \| NGAL (ng/mL) \| \| --- \| | 0.856 | 0.118 | 0.238 | 0.033 | 7.259 | 0.000000 |

*Multiple regression using log iFGF23 as dependent variable as a function serum NGAL and GFR in 48 patients with Chronic Kidney Disease. Model significant at p=1.2E^-13^, R=0.86, R^2^=0.73, Adjusted R^2^=0.72.*
